# Discovery and Validation of DNA Hypomethylation Biomarkers for Liver Cancer Using HRM-Specific Probes

**DOI:** 10.1371/journal.pone.0068439

**Published:** 2013-08-07

**Authors:** Barbara Stefanska, Aurelie Bouzelmat, Jian Huang, Matthew Suderman, Michael Hallett, Ze-Guang Han, Mamun Al-Mahtab, Sheikh Mohammad Fazle Akbar, Wasif Ali Khan, Rubhana Raqib, Moshe Szyf

**Affiliations:** 1 Department of Pharmacology and Therapeutics, McGill University, Montreal, Quebec, Canada; 2 Shanghai-MOST Key Laboratory for Disease and Health Genomics, Chinese National Human Genome Center at Shanghai, Shanghai, Shanghai, China; 3 McGill Centre for Bioinformatics, McGill University, Montreal, Quebec, Canada; 4 Department of Hepatology, Bangabandhu Sheikh Mujib Medical University, Dhaka, Dhaka District, Bangladesh; 5 Department of Medical Sciences, Toshiba General Hospital, Tokyo, Kanto, Japan; 6 International Centre for Diarrhoeal Diseases Research, Bangladesh (icddr,b), Dhaka, Dhaka District, Bangladesh; 7 Sackler Program for Psychobiology and Epigenetics at McGill University, McGill University, Montreal, Quebec, Canada; University of North Carolina School of Medicine, United States of America

## Abstract

Poor prognosis of hepatocellular carcinoma (HCC) associated with late diagnosis necessitates the development of early diagnostic biomarkers. We have previously delineated the landscape of DNA methylation in HCC patients unraveling the importance of promoter hypomethylation in activation of cancer- and metastasis-driving genes. The purpose of the present study was to test the feasibility that genes that are hypomethylated in HCC could serve as candidate diagnostic markers. We use high resolution melting analysis (HRM) as a simple translatable PCR-based method to define methylation states in clinical samples. We tested seven regions selected from the shortlist of genes hypomethylated in HCC and showed that HRM analysis of several of them distinguishes methylation states in liver cancer specimens from normal adjacent liver and chronic hepatitis in the Shanghai area. Such regions were identified within promoters of neuronal membrane glycoprotein M6-B (GPM6B) and melanoma antigen family A12 (MAGEA12) genes. Differences in HRM in the immunoglobulin superfamily Fc receptor (FCRL1) separated invasive tumors from less invasive HCC. The identified biomarkers differentiated HCC from chronic hepatitis in another set of samples from Dhaka. Although the main thrust in DNA methylation diagnostics in cancer is on hypermethylated genes, our study for the first time illustrates the potential use of hypomethylated genes as markers for solid tumors. After further validation in a larger cohort, the identified DNA hypomethylated regions can become important candidate biomarkers for liver cancer diagnosis and prognosis, especially in populations with high risk for HCC development.

## Introduction

Aberrations in epigenetic modifications, particularly in DNA methylation patterns, have been linked to cancer development and progression in many studies in last decades [1], [2], [3]. Hypermethylation of tumor suppressor genes linked to transcriptional silencing, global DNA demethylation associated with genome rearrangements and instability, and recently reported promoter hypomethylation linked to activation of oncogenes and prometastatic genes are hallmarks of nearly all types of cancer [2], [3], [4], [5]. DNA hypermethylation of tumor suppressor genes has been shown to have diagnostic potential in several cancers [6], [7], [8], however our recent unraveling of the broad scope of hypomethylation in liver cancer suggested that potential biomarkers might be found in hypomethylated genes that play a critical role in driving cancer and cancer metastasis [4]. Identifying reliable biomarkers of HCC is of particular importance since the late onset of clinical symptoms accounts for a late diagnosis and high mortality rate. It is estimated that early detection of HCC increases cure rate from 5% to 80% [9].

We previously used a genome-wide approach to delineate DNA promoter methylation profiles in HCC tumors and revealed nearly 2,000 genes whose promoters were hypomethylated in tumors compared with matched adjacent normal tissue [4]. These genes were implicated in biological processes and pathways crucial for cancer development and invasion, which points to an important functional role of the observed alterations. Hypomethylation was observed in several gene families across chromosomes. The question arose whether it will specifically mark tumors and differentiate tumor samples from healthy tissue. In our previous work, we focused on comparison between differences in average DNA methylation across the entire promoter and anti-correlated changes in gene expression. We analyzed in detail 230 genes that were hypomethylated and induced in HCC tumors. The majority of these genes fell into a category of promoters with high CpG content. In the present study on DNA hypomethylation biomarkers for liver cancer, we first selected genes that were heavily hypomethylated in HCC samples as compared with matched adjacent normal tissue (≥2-fold change in promoter methylation based on the microarray analysis, *P*<10E-4). This group of genes was then screened for the most consistently hypomethylated probe (200–400 bp region, ≥1.5-fold change, *P*<10E-3) across the patients as determined by the microarray analysis. In order to increase the functional relevance of potential biomarkers, we subsequently limited this list to 47 genes that were induced in HCC tumors as measured by Affymetrix arrays (≥1.5-fold change, *P*<0.05, Table S1). Taking into account changes in promoter methylation, expression, and the extent of demethylation of the specific probes, we have chosen seven genes that demonstrated the highest promoter hypomethylation, the most hypomethylated probes across the patients and high induction of expression. In addition, we limited the list to genes with cancer-related functions based on publicly available datasets as detailed below. These seven hypomethylated genes as listed in Table 1 were selected to identify and optimize specific probes whose differential methylation would distinguish tumors from normal tissues using High Resolution Melting analysis (HRM). The HRM analysis is a simple PCR-based method easily translatable to clinical setting for detection of differences in DNA methylation patterns [10]. This method requires treatment of DNA with bisulfite that converts cytosine residues to uracil leaving 5-methylcytosine residues unaffected. Following amplification, it results in changes in DNA sequence where uracil is converted to thymidine and 5-methylcytosines remain as cytosines. Unmethylated DNA amplicon that contains thymidine instead of cytosine within CpG sequences exhibits different melting properties than methylated DNA. Following primer optimization, three probes corresponding to three genes *NEURONAL MEMBRANE GLYCOPROTEIN M6-B* (*GPM6B*), *MELANOMA ANTIGEN FAMILY A 12* (*MAGEA12*), and *IMMUNOGLOBULIN SUPERFAMILY FC RECEPTOR* (*FCRL1*), were found to differentiate HCC from adjacent normal tissue and/or invasive tumors from non-invasive tumors in a Chinese set of samples as well as HCC from chronic hepatitis B infection (CHB) in samples from Bangladesh. *GPM6B* is involved in neural development and regulation of bone formation [11], [12]. Expression of this gene was shown in genome wide transcriptome profiling to serve as a predictor of brain tumors [13], [14] and lymphoid leukemias [15]. High expression of *MAGEA12*, a member of oncogenic *MAGE* family, was shown to be associated with bladder carcinoma [16], melanoma [17], breast cancer [18] and oral squamous cell carcinoma [19]. *FCRL1* is a cell-surface membrane protein preferentially expressed on B cells regulating B cell activation and differentiation [20]. High expression of this gene was observed in metastatic melanomas [21] and in different types of leukemias [20]. None of these genes or their state of methylation was previously linked to HCC. *MAGEA12* was shown before to be repressed in normal cells by promoter methylation and activated in cancer cells by demethylation [22], [23], whereas the role of *GPM6B* and *FCRL1* methylation in promoter activity was not previously tested. Our present study validates for the first time overexpression of *GPM6B*, *MAGEA12* and *FCRL1* in HCC patients relative to normal tissue and investigates DNA methylation within their promoters as a potential diagnostic marker of liver cancer. Since early diagnosis of HCC increases the cure rate and survival, the identified epigenetic candidate biomarkers could have an impact on liver cancer therapy outcome after validation in a larger cohort.

**Table 1:** Functional analysis of top 7 genes whose promoters were significantly hypomethylated in liver cancer samples compared to matched adjacent normal tissue as assessed by genome-wide promoter microarray.

Gene symbol	Gene name	Chromosome	Pathway/Function/Biological process
DLGAP5	discs, large (Drosophila) homolog-associated protein 5	14	Hepatoma up-regulated protein; regulation of cell cycle, cell adhesion, cell-cell signaling, proliferation and differentiation; positive regulation of mitotic metaphase/anaphase transition; phosphoprotein phosphatase activity
FCRL1	immunoglobulin superfamily Fc receptor, gp42	1	Regulation of cell proliferation, B-cell activation and differentiation; regulation of cancer cell growth; receptor activity
GPM6B	neuronal membrane glycoprotein M6-b	X	Involved in neural development, regulation of osteoblast function and bone formation, matrix vesicle release by osteoblasts; maintenance of the actin cytoskeleton; negative regulation of serotonin uptake
MAGEA12	melanoma antigen family A, 12; cancer/testis antigen family 1, member 12	X	Not very well known; May play a role in transformation and tumor progression; in vitro increases viability of cancer cells
MMP1	matrix metallopeptidase 1 (interstitial collagenase)	11	Involved in the breakdown of extracellular matrix, blood coagulation, leukocyte migration, collagen catabolic process and cancer metastasis
SSX1	sarcoma, synovial, X-chromosome-related 1; cancer/testis antigen family 5, member 1	X	Regulation of transcription; transcription corepressor activity; involved in humoral and cellular immune responses in cancer patients; involved in the t(X;18) translocation found in all synovial sarcomas (fusion of the synovial sarcoma translocation gene on chromosome 18 to one of the SSX gene on chromosome X, the rsulting hybrid is responsible for transforming activity)
TPO	thyroid peroxidase	2	Involved in thyroid gland function, thyroid hormone generation, embryonic hemopoiesis

Functional analyses were performed using GO database.

## Materials and Methods

### Ethics statement

All patients provided written informed consent, and the Ethics Committee from concerned institutions (Chinese National Human Genome Center at Shanghai, China and Bangabandhu Sheikh Mujib Medical University (BSMMU), Dhaka, Bangladesh) approved the study.

### Patients and tissue samples

Cancerous and normal adjacent tissue samples were obtained from 12 patients with HCC and 1 patient with inflammatory pseudotumor (control patient) in Chinese National Human Genome Center at Shanghai, China (Dr. Ze-Guang Han) (Table 2). Bangladeshi cohort consisted of 4 HCC patients and 4 CHB patients in Bangabandhu Sheikh Mujib Medical University (BSMMU), Dhaka, Bangladesh (Table 2). The HCC samples were obtained at ultrasonogram guided fine needle aspiration, while the CHB samples were obtained at per-cutaneous liver biopsy using Tru-cut biopsy needle. In both cases local anaesthesia was used.

**Table 2:** Clinicopathological characteristics of patients from Chinese and Bangladeshi cohorts.

	Patient ID	Gender	Age	Cellular type	Differentiation stage	Portal vein infiltration
Chinese	1	Male	48	HCC	Middle	Yes
	4	Male	31	HCC	Low	Yes
	5	Female	69	HCC	Middle	Yes
	6	Male	51	HCC	Middle	Yes
	7	Male	80	HCC	Middle to low	Yes
	8	Male	43	HCC	High	No
	9	Male	50	HCC	Middle	No
	10	Male	44	HCC	High	No
	11	Male	73	HCC	Middle to low	No
	12	Female	51	HCC	Middle to high	No
	13	Male	40	Inflammatory pseudotumor	-	No
	14	Male	52	HCC	Middle	No
	15	Male	31	HCC	Middle	No
Bangladeshi	1	Male	22	CHB	-	-
	2	Male	48	HCC	-	-
	3	Male	35	CHB	-	-
	4	Male	29	CHB	-	-
	5	Male	35	HCC	-	-
	6	Male	55	HCC	-	-
	8	Male	20	CHB	-	-
	9	Male	34	HCC	-	-

### DNA isolation and bisulfite treatment of DNA

DNA from cancerous, normal adjacent, and CHB samples was isolated using standard phenol-chloroform extraction technique. Bisulfite conversion was performed as previously described [24]. Briefly, 2 µg of DNA was linearized with EcoRI and incubated for 3 h at 37°C followed by purification using the Quick Clean PCR Purification Kit (GenScript) according to the manufacturer's protocol. The purified DNA was then denatured with 3M NaOH and incubated for 15 minutes at 37°C. Freshly prepared 3.6M sodium bisulfite/1 mM hydroquinone mixture (pH 5.0) was added to denatured DNA and incubated for 2 minutes at 95°C and then 8 h at 55°C followed by 2 minutes at 95°C and 2 h at 55°C. DNA samples were then desalted and purified (Quick Clean PCR Purification Kit, GenScript). The purified DNA was again denatured with 3M NaOH and incubated for 15 minutes at 37°C. The solution was neutralized by addition of ammonium acetate to a final concentration of 10M and the DNA was precipitated with 95% ethanol and the pellet was resuspended in 50 µl of distilled water.

### PCR amplification of bisulfite converted DNA

Amplification reactions contained 25 µg of bisulfite-treated genomic DNA, 0.4 µM forward and reverse primers listed in Table S2, 10 µl of 10× Light Cycler 480 SybrGreen I Master (Roche) in a final volume of 20 µl. Amplification was performed in a thermocycler using the following conditions: denaturation at 95°C for 10 min, amplification for 40 cycles at 95°C for 1 min, annealing temperature for 2 min 30 s, 72°C for 1 min, and final extension at 72°C for 5 min. As shown in Table S2, outside and nested primer sets were used for several probes in order to improve the efficiency of amplification. The amplified DNA was then subjected to high resolution melting (HRM).

### High resolution melting analysis (HRM)

The amplified DNA was transferred to the Light Cycler 480 QPCR instrument (Roche) that enables HRM. Samples were gradually heated from 40°C for 1 min to 60°C for 15 s and finally DNA was melted at 95°C for 15 s. The melting of the PCR product induces a decrease in the fluorescence of SybrGreen since SybrGreen as a DNA intercalating dye is being released from double-stranded DNA following dissociation. The fluorescence signal is acquired during the melting phase and analyzed by the Light Cycler 480 software. The temperature of a melting peak of DNA amplicon is defined by the midpoint of the melt phase at which the rate of changes in fluorescence is the largest. This melting point depends on the sequence and the length of the amplicon and is specific to each product. As following bisulfite conversion methylated DNA after amplification contains CG base pairs, the melting temperature of an unmethylated version that contains TA base pairs is different. The methylated amplicon melts at higher temperature than when unmethylated and samples of different methylation state can be separated by comparing the melting curve peaks. Using 0% and 100% methylated control DNA, we showed that the tested probes reveal clear differences between unmethylated and fully methylated DNA. 0% methylated control was generated using whole genome amplification kit (Sigma), whereas 100% control was created by *in vitro* methylation reaction catalyzed by SssI methyltransferase in the presence of a methyl donor S-adenosyl-L methionine (SAM). Melting curve peaks in HCC tumors were compared with the average of melting peaks in normal adjacent tissues from 7 individuals with non-invasive HCC. This average curve was called here as normal adjacent reference (NorAdjRef).

### Pyrosequencing

Specific bisulfite converted promoter sequences were amplified with HotStar Taq DNA polymerase (Qiagen) using biotinylated primers listed in Table S2. The biotinylated DNA strands were pyrosequenced in the PyroMarkTMQ24 instrument (Biotage, Qiagen) as previously described [25]. Data were analyzed using PyroMarkTMQ24 software.

### RNA extraction and Quantitative real-time PCR (QPCR)

Total RNA was isolated using TRIzol (Invitrogen, Life Technologies, Carlsbad, CA, USA) according to the manufacturer's protocol. 1 µg of total RNA served as a template for cDNA synthesis using 20 U of AMV reverse transcriptase (Roche Diagnostics), as recommended by the manufacturer. The QPCR reaction was carried out in Light Cycler 480 machine (Roche) using 2 µl of cDNA, 400 nM forward and reverse primers listed in Table S2 and 10 µl of Light Cycler 480 SybrGreen I Master (Roche) in a final volume of 20 µl. Amplification was performed using the following conditions: denaturation at 95°C for 10 min, amplification for 60 cycles at 95°C for 10 s, annealing temperature for 10 s, 72°C for 10 s, and final extension at 72°C for 10 min. Quantification was performed using a standard curve and analyzed by the Roche LightCycler 480 software.

### Statistical analysis

Statistical analysis was performed using unpaired two-sided *t*-test or Mann-Whitney *U* test. The Mann-Whitney *U* test was used for comparisons between two groups where sample sizes were small and it was therefore difficult to verify distributional assumptions. In each case, the test is accompanied by a scatter plot showing all values used in the test. Mean values are given ± S.D. Where necessary, *P*-values are adjusted for multiple tests using Bonferroni correction. For comparisons of four vs. four samples (Bangladeshi cohort), we also used a permutation test where the data were shuffled 10,000 times and the test statistic was recalculated for each shuffle. The R software environment for statistical computing was used for all statistical analyses except the permutation test where the Resampling Stats Add-in for Excel software was used (Stan Blank, Charles Seiter, Peter Bruce and Resampling Stats, Inc. 2000, the Institute for Statistics Education, USA).

## Results

### Testing and identification of candidate hypomethylated regions in HCC by HRM analysis

**Figure 1:**
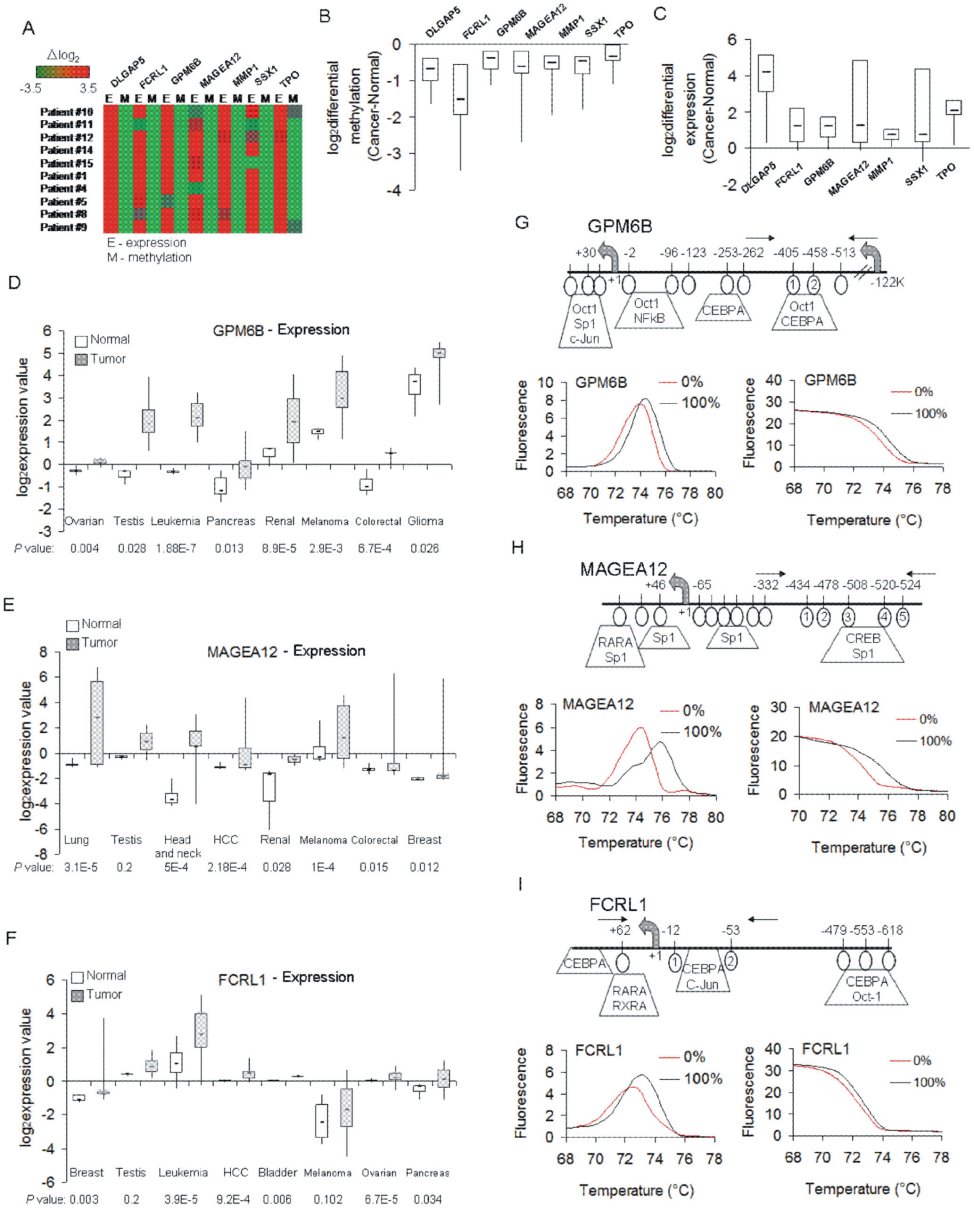
Hypomethylated and activated genes in HCC patients tested for biomarkers; characterization of optimized HRM probes corresponding to *GPM6B*, *MAGEA12*, and *FCRL1*. (A) A heatmap showing log_2_ differential methylation (M) and expression (E) probe intensities for the indicated seven genes in every HCC patient samples as measured by microarrays. (B,C) Box plots of these methylation (B) and expression (C) differences in HCC patients (log_2_ [cancer-normal]). Negative methylation differences indicate hypomethylation in HCC and positive expression differences reflect higher expression in tumor samples as compared with matched adjacent normal tissue. (D,E,F) Cancer gene expression levels obtained from microarray data for *GPM6B* (D), *MAGEA12* (E), and *FCRL1* (F). The normal versus tumor gene expression data were obtained from Oncomine and are presented as log_2_-transformed median centered per array, and SD-normalized to 1 per array. All the presented changes are statistically significant (*P*<0.05) except *MAGEA12* in testicular cancer and *FCRL1* in testicular cancer and melanoma. (G,H,I) Melting curves and melting curve peaks for 0% and 100% methylated control DNA amplified for *GPM6B* (G), *MAGEA12* (H) and *FCRL1* (I) along with a map of promoters of the tested genes flanking the probes selected for HRM analysis. Horizontal arrows indicate the position of primers used for HRM. HRM for 0% and 100% methylated control DNA are shown. CpG sites that are located within the amplified probe are circled and numbered. The putative transcription factor binding sites are indicated as predicted by TransFac.

The goal of our study was to identify and optimize hypomethylated regions in HCC that could differentiate HCC from normal tissue using HRM. We applied several criteria that we predicted will increase the probability that the probes are ubiquitously differentially methylated in HCC. First, we selected seven genes (listed in Table 1) that were identified in our previous screen of hypomethylated genes in liver cancer using methylated DNA immunoprecipitation (MeDIP) and hybridization to genome-wide promoter oligonucleotide arrays. Second, the gene promoters contained probes with an average fold reduction in methylation between HCC and normal liver higher than or equal 2 and were highly statistically significant (*P*<10E-4). Third, hypomethylation of their promoters was also associated with overexpression in almost all the patients that were studied suggesting robustness and functional relevance of these DNA demethylation changes in HCC (Figure 1A–C). Fourth, the analysis of their functions based on publicly available datasets such as Gene Ontology (GO), KEGG, and NCBI reveals biological processes and pathways that are crucial for carcinogenesis such as regulation of cell cycle, cell adhesion, cell-cell signaling, proliferation and differentiation (Table 1). Fifth, the genes were previously associated with human cancers, in particular *MAGEA12* [16], [17], [18], [19] however only *DISCS LARGE (DROSOPHILA) HOMOLOG-ASSOCIATED PROTEIN 5* (*DLGAP5*) and *MATRIX METALLOPEPTIDASE 1* (*MMP1*) have been previously reported to be up-regulated in HCC tumors [26], [27]. Sixth, using publicly available expression microarray data (Oncomine), we demonstrate that three of the genes that contained probes optimized for HRM analysis (see paragraph below) show increased expression in many different cancer types such as leukemia, ovarian and pancreatic cancer (*GPM6B* and *FCRL1*), renal and colorectal cancer (*GPM6B* and *MAGEA12*), HCC and breast cancer (*MAGEA12* and *FCRL1*), testicular cancer and melanoma (significant overexpression of *GPM6B* in both cancers and *MAGEA12* in melanoma) (Figure 1D–F). The data are consistent with a general role for these proteins in many different types of cancer and suggest their fundamental role in cancer progression and/or metastasis.

### Three probes corresponding to *GPM6B*, *MAGEA12*, and *FCRL1* optimized for HRM analysis of differential methylation in HCC

Several methylation-independent primer sets were subjected to optimization for HRM analysis (see primer sequences in Table S2). Control DNA that was methylated at 0% and 100% (standards) was amplified with the primers and melted in real-time. The melting pattern of the amplicons was established and analyzed. Melting curves for both standards were plotted in the same chart and exhibited different temperatures of melting peaks (Figure 1G–H). We then screened 12 HCC and 12 normal liver samples from the Shanghai cohort for methylation state within all 7 probes using designed primers. Four probes exhibited high heterogeneity of the methylation pattern in tumors, which was reflected in multiple melting peaks in HRM analysis for a single sample. Three other probes for *GPM6B*, *MAGEA12*, and *FCRL1* showed a clear one-peak melting pattern and were used for further analyses as candidate DNA methylation markers. Overexpression of these genes was shown in other cancers, for instance higher expression of *GPM6B* was associated with brain tumors [13], [14] and lymphoid leukemias [15], *MAGEA12* was up-regulated in bladder carcinoma [16], melanoma [17], breast cancer [18] and oral squamous cell carcinoma [19], whereas *FCRL1* induction was observed in metastatic melanomas [21] and in different types of leukemias [20].

### Differential HRM pattern of amplicons in *GPM6B*, *MAGEA12*, and *FCRL1* differentiates HCC from normal liver

**Figure 2:**
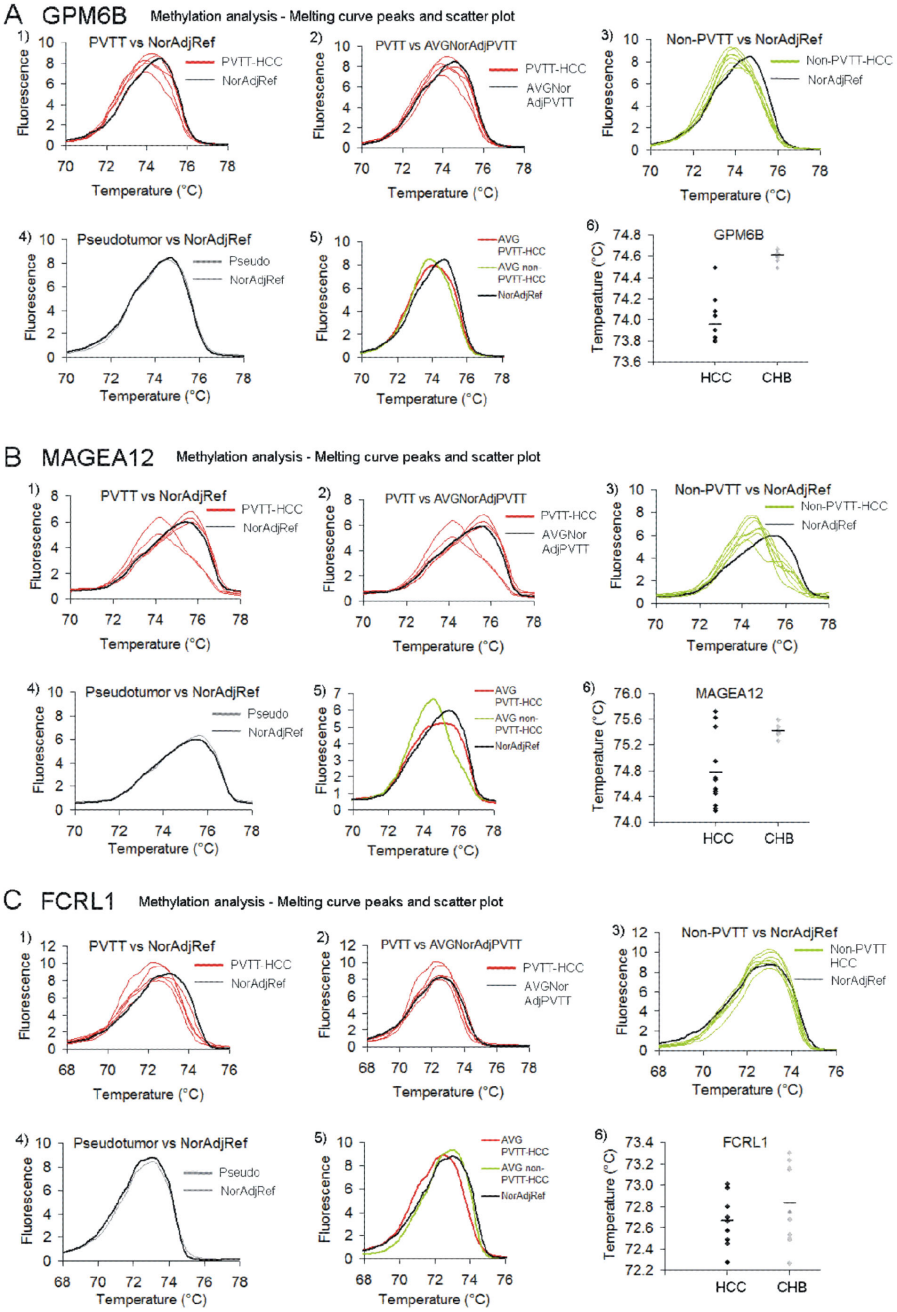
Methylation levels of *GPM6B*, *MAGEA12* and *FCRL1* promoters in HCC and non-cancerous tissue as measured by HRM. (A–C) Melting curve peaks of *GPM6B* (A), *MAGEA12* (B) and *FCRL1* (C) amplicons in 12 tumor samples and 12 matched adjacent normal tissues. The peaks for HCC with PVTT (PVTT-HCC), HCC without PVTT (non-PVTT-HCC), and pseudotumor subjects were compared with the average of normal adjacent tissues from non-PVTT patients (NorAdjRef). In the second panel, melting peaks for tumor samples of HCC patients with PVTT were plotted together with the average of matched adjacent normal tissues from these patients, whereas the fifth panel shows the average of HCC with PVTT and HCC without PVTT in comparison with NorAdjRef. Temperature values for melting peaks in every patient are shown in scatter plots and in Table 3.

**Figure 3:**
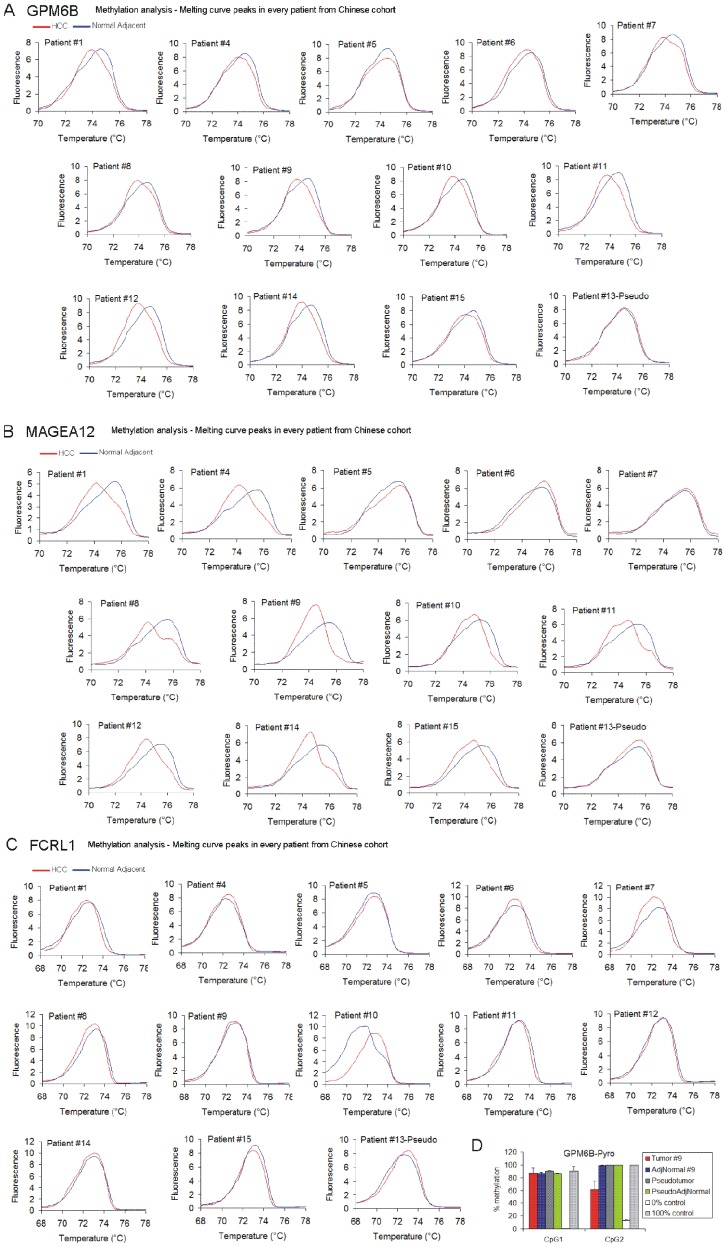
Methylation levels of *GPM6B*, *MAGEA12* and *FCRL1* promoters in individual HCC patients from the Chinese cohort as measured by HRM in comparison with the matched adjacent normal tissue from the same patient. (A–C) Melting curve peaks of *GPM6B* (A), *MAGEA12* (B) and *FCRL1* (C) amplicons in tumor samples in comparison to matched adjacent normal tissue from the same patient. (D) Pyrosequencing of *GPM6B* region for patient 9 (HCC) and patient 13 (pseudotumor). The covered region overlaps with the sequence analyzed in HRM.

HRM analysis of tumors (n = 12) and adjacent normal tissues (n = 12) was performed. The melting curves were plotted and the temperature of a melting peak was calculated as described in the methods. As the methylated DNA amplicon melts at higher temperature than amplicons from unmethylated bisulfite converted DNA, samples of different methylation states can be differentiated by comparing the melting curve peaks. It is a common practice to compare tumors with pathologically normal adjacent tissue from the same patient. However, it may result in false negative results since so called normal liver can be already transformed molecularly and displayed altered DNA methylation patterns without exhibiting changes in histopathology, particularly in patients with highly invasive tumors. Five patients in the sample set were diagnosed with invasive HCC where portal vein tumor thrombus (PVTT) was detected. We assumed that normal adjacent liver can be already changed in these patients. We therefore created a normal reference curve (NorAdjRef) by averaging melting curves for all adjacent normal tissues excluding patients with PVTT. We suggest that such a reference curve could be further improved by including HRM results from a large sample of non-cancerous liver tissue and be used as a general standard for comparison for new cases particularly when invasion into adjacent tissue is suspected. Indeed, methylation of *GPM6B* probe in the tumor sample from patient 5 was the same as in its adjacent normal tissue, however it was different from NorAdjRef confirming the use of an averaged normal as a standard for comparison (Figure 2A, Figure 3A). Patients 1–6 with PVTT exhibited the same methylation state within *FCRL1* probe when their tumors were compared to their matched adjacent normal tissue but again the HRM profiles were different from NorAdjRef (Figure 2C). Thus, by using an averaged normal standard we were able to call these samples as HCC, which would have been impossible if we used the adjacent tissue as a reference.

We have optimized three specific 150 bp DNA regions that could differentiate HCC from untransformed tissue using HRM. A region within *GPM6B* promoter was hypomethylated in tumors of all patients as compared with NorAdjRef (Figure 2A). All tumors were clearly separated and identified as HCC based on temperature values of melting peaks (Table 3, *P* = 3.97E-5, adjusted *P* = 1.17E-4, Mann-Whitney *U* test). Subjects with non-invasive HCC (non-PVTT) were more hypomethylated compared to invasive tumors as the difference in melting peaks between cancer and NorAdjRef indicates. Thus, the amplicon could differentiate also HCC without PVTT from HCC with PVTT, at least in the small number of cases examined here (Table 3, Figure 2A-charts 3 and 5, *P* = 0.03, adjusted *P* = 0.06, Mann-Whitney *U* test). HRM analysis of a region within *MAGEA12* promoter differentiated all HCC without PVTT and 2 out of 5 HCC with PVTT from NorAdjRef (Figure 2B, Figure 3B, *P* = 1.7E-4, adjusted *P* = 5.1E-4, Mann-Whitney *U* test). Tumor samples from patients 5, 6, and 7 who were diagnosed with PVTT exhibited the same methylation level as their matched normal adjacent tissue (Figure 2B-chart 2, Figure 3B) and were not distinguished either from NorAdjRef (Figure 2B-chart 1). The average melting peak temperature value for the HCC without PVTT was lower than for the average HCC with PVTT indicating hypomethylation within *MAGEA12* probe in non-invasive tumors (Table 3, Figure 2B-chart 5), although the change was not statistically significant in our set of samples (*P* = 0.64, Mann-Whitney *U* test). HRM for the *FCRL1* probe differentiates HCC with PVTT from NorAdjRef (*P* = 2.5E-3, adjusted *P* = 7.5E-3, Mann-Whitney *U* test) but not HCC without PVTT from NorAdjRef (*P* = 0.11, Mann-Whitney *U* test) (Figure 2C-chart 1). The *FCRL1* probe is hypomethylated in invasive compared to non-invasive tumors (Table 3, Figure 2C-chart 5, *P* = 2.5E-3, adjusted *P* = 7.5E-3, Mann-Whitney *U* test) and additionally differentiates these two kinds of tumors. Interestingly, although we nicely differentiate by HRM PVTT samples from NorAdjRef, there was no relevant difference when we compared *FCRL1* methylation state in every PVTT tumor sample with the matched adjacent normal tissue from the same patient except patient 7 (Table 3, Figure 2C-chart2, Figure 3C). This suggests that the DNA methylation changes occurred already in the adjacent tissue in these livers. Patient 13 who was diagnosed with liver pseudotumor showed no difference in the HRM melting profile with its own adjacent tissue and NorAdjRef in all three probes (Table 3, Figure 2-charts 4, *P*>0.05). We confirmed this observation by pyrosequencing of *GPM6B* region for HCC subject 9 and pseudotumor patient as shown in Figure 3D.

**Table 3:** Temperature values of melting curve peaks of *GPM6B* (A), *MAGEA12* (B) and *FCRL1* (C) amplicons in tumor samples and normal liver tissues in Chinese (Ch) and Bangladeshi (B) cohorts as analyzed by HRM.

		GPM6B					MAGEA12					FCRL1				
		HCC		NorAdj/CHB			HCC		NorAdj/CHB			HCC		NorAdj/CHB		
		AVG	SD	AVG	SD	*P*	AVG	SD	AVG	SD	*P*	AVG	SD	AVG	SD	*P*
Ch	Patient 1 (PVTT)	73.91	0.04	74.62	0.10	<0.0001	74.21	0.13	75.41	0.21	<0.0001	72.46	0.22	72.51	0.35	0.016
	Patient 4 (PVTT)	74.04	0.05	74.59	0.15	<0.0001	74.18	0.02	75.38	0.26	<0.0001	72.49	0.17	72.27	0.19	0.018
	Patient 5 (PVTT)	74.49	0.01	74.49	0.01	0.018	75.62	0.01	75.40	0.15	0.166	72.49	0.18	72.54	0.20	0.018
	Patient 6 (PVTT)	74.19	0.13	74.56	0.11	0.0002	75.48	0.21	75.48	0.11	0.58	72.58	0.05	72.49	0.17	0.031
	Patient 7 (PVTT)	73.84	0.06	74.60	0.06	<0.0001	75.72	0.03	75.60	0.05	0.06	72.28	0.27	72.68	0.00	0.0056
	Patient 8	73.80	0.01	74.63	0.08	<0.0001	74.26	0.20	75.43	0.29	0.0002	72.97	0.03	73.16	0.10	<0.0001
	Patient 9	73.83	0.14	74.63	0.08	<0.0001	74.49	0.02	75.42	0.32	0.0006	72.58	0.54	72.75	0.68	0.084
	Patient 10	73.80	0.01	74.63	0.01	<0.0001	74.95	0.20	75.26	0.04	0.032	72.66	0.51	73.23	0.00	0.13
	Patient 11	73.81	0.11	74.63	0.08	<0.0001	74.70	0.02	75.40	0.24	0.0027	72.98	0.02	73.30	0.00	0.61
	Patient 12	73.80	0.19	74.67	0.04	<0.0001	74.45	0.07	75.43	0.20	0.0005	73.01	0.02	73.16	0.20	0.73
	Patient 14	73.91	0.06	74.63	0.08	<0.0001	74.52	0.03	75.40	0.24	0.0008	72.80	0.71	72.75	0.64	0.36
	Patient 15	74.08	0.08	74.63	0.08	<0.0001	74.66	0.03	75.37	0.19	0.0021	72.70	0.56	73.15	0.12	0.17
	All HCC Average	73.96	0.03	74.61	0.00	<0.0001	74.77	0.03	75.41	0.19	<0.0001	72.66	0.26	72.83	0.22	0.0028
	Non-PVTT Average	73.86	0.06	74.64	0.06	<0.0001	74.57	0.06	75.39	0.21	<0.0001	72.81	0.33	73.07	0.25	0.6
	Pseudo 13	74.56	0.11	74.53	0.06	0.23	75.59	0.03	75.43	0.20	0.22	72.94	0.27	72.61	0.15	0.53
B	Patient 2 (HCC)	74.09	0.12	-	-	0.47	73.15	0.06	-	-	<0.0001	72.02	0.17	-	-	0.0086
	Patient 5 (HCC)	73.74	0.07	-	-	0.03	73.22	0.06	-	-	<0.0001	71.95	0.07	-	-	0.0002
	Patient 6 (HCC)	73.78	0.02	-	-	0.03	74.36	0.21	-	-	0.001	71.93	0.16	-	-	0.0028
	Patient 9 (HCC)	73.64	0.03	-	-	0.015	73.26	0.11	-	-	<0.0001	72.10	0.11	-	-	0.0046
	HCC Average	73.81	0.00	-	-	0.0053	73.50	0.11	-	-	<0.0001	72.00	0.05	-	-	<0.0001
	Patient 1 (CHB)	-	-	74.12	0.17	-	-	-	75.74	0.18	-	-	-	72.13	0.08	-
	Patient 3 (CHB)	-	-	74.15	0.22	-	-	-	74.50	0.70	-	-	-	72.45	0.28	-
	Patient 4 (CHB)	-	-	74.19	0.27	-	-	-	75.56	0.03	-	-	-	72.25	0.01	-
	Patient 8 (CHB)	-	-	74.33	0.07	-	-	-	75.67	0.08	-	-	-	72.69	0.25	-
	CHB Average	-	-	74.20	0.18	-	-	-	75.36	0.10	-	-	-	72.38	0.01	-

The value for every patient in Chinese cohort was compared with the average of normal adjacent tissue in 7 non-invasive patients (NorAdjRef, non-PVTT average). The value for every patient in Bangladeshi cohort was compared with the average of all CHB samples.

In summary, a combination of these three HRM optimized probes would differentiate tumors from non-tumors (using *GPM6B* probe and *MAGEA12* in combination with *GPM6B*) and HCC with PVTT from non-PVTT HCC (using *FCRL1* probe).

### Validation of identified epigenetic biomarkers in Bangladeshi biopsy specimens from HCC and chronic hepatitis B (CHB) patients

**Figure 4:**
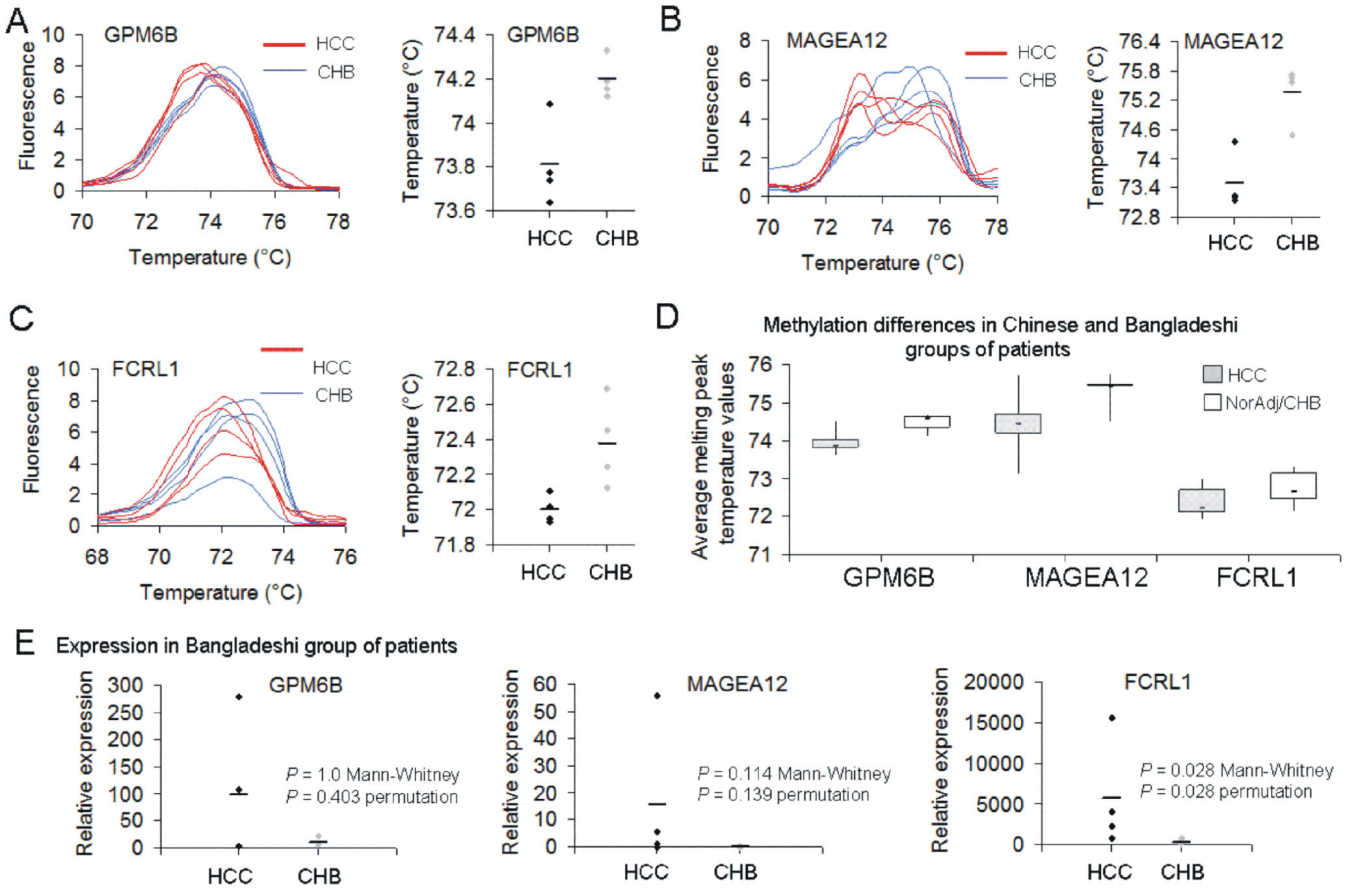
Methylation and expression levels of *GPM6B*, *MAGEA12* and *FCRL1* in HCC and chronic hepatitis B (CHB) patients as measured by HRM and QPCR, respectively. Melting curve peaks of *GPM6B (A)*, *MAGEA12* (B) and *FCRL1* (C) amplicons in 4 HCC and 4 CHB patients from Bangladesh. Temperature values for melting peaks in every patient are shown in scatter plots and in Table 3. (D) Box plots of the average melting peak temperature values across all the patients in both cohorts. Lower temperature value indicates lower methylation level within a probe. (E) Relative expression of the tested genes in HCC and CHB subjects as measured by QPCR and depicted as scatter plots. *P* values calculated using Mann-Whitney *U* test and permutation test are shown.

The critical question in diagnosis of HCC is identifying early conversion of CHB to HCC. We therefore asked whether the identified probes could distinguish between HCC and CHB in an independent set of subjects. We tested 4 subjects with HCC and 4 subjects with CHB (Table 2 for clinical characteristics). The *GPM6B* probe which differentiated all HCC from NorAdjRef in the Shanghai group of patients also showed different melting patterns in HCC and CHB except one patient with HCC who was grouped together with CHB based on melting peak temperature (Table 3, Figure 4A, *P* = 0.028, adjusted *P* = 0.084, Mann-Whitney *U* test; *P*<0.0001, permutation test). Methylation state within *MAGEA12* probe separated all HCC cases from CHB as indicated by lower melting temperature (Table 3, Figure 4B, *P* = 0.028, adjusted *P* = 0.084, Mann-Whitney *U* test; *P* = 0.02, permutation test). In HCC patients, we clearly observed two melting peaks indicating two populations of cells with low and high *MAGEA12* methylation level. A probe within *FCRL1* was hypomethylated in all tumors as compared with the average of all CHB patients (Table 3, Figure 4C, *P* = 0.028, adjusted *P* = 0.084, Mann-Whitney *U* test; *P*<0.0001, permutation test). When we grouped melting peak temperature values for all HCC samples and all adjacent normal and CHB samples, median value for each tested probe was lower in cancer than in normal indicating hypomethylation in cancer (Figure 4D). The differences were highly statistically significant for *GPM6B* and *MAGEA12* probes (*P*<0.001, adjusted *P*<0.003, Mann-Whitney *U* test) as well as for *FCRL1* but after excluding HCC samples without PVTT (*P*<0.01, adjusted *P*<0.03, Mann-Whitney *U* test). Expression of the tested genes as measured by QPCR in the Bangladeshi group was on average higher in HCC than in CHB subjects (see Figure 4E for exact values in every patient) with the most relevant changes observed for *FCRL1* (Figure 4E, *P* = 0.028, adjusted *P* = 0.084, Mann-Whitney *U* test; *P* = 0.028, permutation test).

## Discussion

Late onset of clinical symptoms of HCC accounts for late diagnosis and poor prognosis. As early detection of HCC improves cure rate from 5% to 80%, there is a need for development of diagnostic biomarkers [9]. Epigenetic alterations, especially changes in DNA methylation patterns, have been implicated in many types of human cancer and observed at initiation and progression stages [1], [2], [3]. Recent genome-wide approaches delineated cancer-specific changes in DNA methylation that open the door to the emerging field of DNA methylation markers for early detection, prognosis and response to therapy. For instance, DNA methylation within paired-like *Homeodomain Transcription Factor 2* (*PITX2*) has been validated using paraffin-embedded tissues as a predictive marker of adjuvant tamoxifen therapy outcome in breast cancer patients [6]. Our recent studies revealed robust changes in the landscape of DNA methylation in HCC patients and showed that promoter specific hypomethylation is associated with activation of cancer-driving genes [4]. High frequency of DNA promoter hypomethylation was subsequently confirmed in another study on different sets of HCC samples [28] as well as in neuroblastoma samples [5]. These studies highlight the importance of promoter hypomethylation in cancer and the need to further explore the role of demethylation in cancer. One practical question is whether genes that are targets of hypomethylation in cancer can serve as markers that could improve early diagnosis and/or prognosis.

In the present study, we screened for potential DNA hypomethylation markers for liver cancer diagnosis among genes that we previously found to be hypomethylated in HCC patients. Our aim was to develop primers for specific probes within these genes that will differentiate HCC from normal liver, chronic hepatitis as well as PVTT invasive HCC using a simple PCR based method that could easily be translated into clinical settings. We have identified three specific DNA probes covering regions around 150 bp long as potential candidates for markers in HCC using a simple PCR-based HRM method. The probes correspond to three genes *GPM6B*, *MAGEA12*, and *FCRL1* that were overexpressed and demethylated in our set of HCC patients (Figure 1B and 1C) and up-regulated in many other types of cancer (Figure 1D–F). Differential methylation of promoters of these genes was not previously reported in cancer although methylation-dependent regulation of *MAGEA12* promoter was shown in several cancer cell lines [23].

As a reference in our analyses, we used the average of all adjacent normal tissues from HCC patients without PVTT (non-PVTT HCC) termed as a normal adjacent reference curve (NorAdjRef) rather than the adjacent to tumor liver sample of the same case. The rationale behind this approach is the many false negative results obtained when tumor samples are being compared with pathologically normal adjacent tissue from the same patient. We speculate that the normal tissue, especially in patients with already developed metastases, can be already transformed and bear altered DNA methylation patterns without changes in histology. In our study, it was the case for patient 5 who was identified based on HRM as a cancer subject only when *GPM6B* methylation in the tumor sample was compared with NorAdjRef (Figure 2A, Figure 3A). Similarly, *FCRL1* methylation separated tumors of patients 1–6 from NorAdjRef but not from adjacent tissue from the same patient (Figure 2C). Our approach minimizes false negative results and increases specificity, which might facilitate detection of liver transformation early before the histopathological changes are visualized. Using an average normal curve provides an “objective” standard for comparison for unknown cases. Our results obtained from a limited set of subjects must be validated in a larger clinical study. Our data nevertheless point to the feasibility of this approach. It is particularly important to determine whether using an average non-cancer reference will indeed improve our ability to detect HCC prior to manifestation of histopathological changes.

The *GPM6B* probe differentiated all tested HCC samples either with or without PVTT from NorAdjRef (Table 3, Figure 2A). A probe within *MAGEA12* should be used in combination with GPM6B in order to detect cancer as melting curves for tumors of a few patients with PVTT overlap with NorAdjRef curve (Table 3, Figure 2B). The third probe located within *FCRL1* promoter is hypomethylated in invasive tumors and differentiates invasive from non-invasive cases (Table 3, Figure 2C). The combination of probes should detect all HCC cases and differentiate PVTT and non-PVTT HCC. All three probes distinguished HCC from CHB in Bangladeshi set of samples as compared with the average of CHB melting peaks (Table 3, Figure 4A–C). In both Chinese and Bangladeshi groups of patients, HCC was associated with hepatitis B virus (HBV) infection that is the major cause of primary liver cancer in these countries. The incidence of liver cancer in China and Bangladesh is rising together with HBV infection. The fact that we could validate cancer-specific hypomethylation of the identified probes in a different group of patients from a widely different ethnic background in a different geographical location points to the feasibility of using the identified probes as markers of HCC. High statistical relevance of the differences in melting temperature values (Figure 4D, *P*<0.001 for *GPM6B* and *MAGEA12*, *P*<0.01 for *FCRL1* after excluding HCC without PVTT, Mann-Whitney *U* test) even with the small sample size provides justification for larger studies. Moreover, it will be of interest to test the identified probes in circulating tumor cells in blood as potentially non-invasive source of DNA. There is previous evidence for the feasibility of this approach in other cancers. A *MAGEA* gene expression test that provides information on multiple *MAGEA* genes in a single reaction detected circulating tumor cells in blood of melanoma, breast and colorectal patients [29]. Brennan *et al.* discovered methylation in *ATAXIA TELANGIECTASIA MUTATED* (*ATM*) intragenic loci in DNA from white blood cells as a potential marker of breast cancer risk [30]. Circulating methylated *SEPTIN 9* (*SEPT9*) DNA and *INTERMEDIATE FILAMENT FAMILY ORPHAN 1* (*IFFO1*) DNA in plasma were found to correlate with the occurrence of colorectal cancer [7] and ovarian cancer [8], respectively.

In contrary to most of the DNA methylation marker studies that focus on promoters that are hypermethylated in cancer, we unravel genes and specific probes within their promoters that are hypomethylated in cancer as compared with normal tissue. Using HRM, a straightforward method providing results in a short period of time, the identified probes are highly suitable for further testing for clinical applications and introduction into routine clinical tests after validation in larger studies.

Although our study is limited in the number of cases examined and did not have the necessary power to distinguish early and late stages of liver cancer, it is remarkable that even with a small sample size (cancer: n = 16, normal: n = 16) we were able to obtain highly significant results (Figure 4D). This positions these probes as excellent candidates for a larger clinical study. If proven successful early markers of HCC could be used to predict liver cancer in high-risk populations for developing HCC such as hepatitis B and C infection, type 2 diabetes mellitus, and alcohol-driven cirrhosis.

## Supporting Information

Table S1 **Candidate genes for screening for epigenetic biomarkers of liver cancer.**
(XLS)

Table S2 **Primer sequences and description of all the specific probes that were tested in HRM.**
(DOC)
