# Supplementary material for: Discovery and Validation of DNA Hypomethylation Biomarkers for Liver Cancer Using HRM-Specific Probes
Source: PLoS One. 2013 Aug 7;8(8):e68439. doi: 10.1371/journal.pone.0068439 (PMC3737236; doi:10.1371/journal.pone.0068439)
Supplement: Table S2 — Primer sequences and description of all the specific probes that were tested in HRM. (DOC) [file pone.0068439.s002.doc]

**Supplementary Table S2.** Primer sequences and description of all the specific probes that were tested in HRM.

**A) Outside primers for HRM.**

| **Gene** | **Probe #** | **Region [**UCSC, hg18**]** | **Outside primers** | | | |
| --- | --- | --- | --- | --- | --- | --- |
|  |  |  | **Sequence** | **Position [**UCSC, hg18**]** | **Amplicon size [bp]** | **Tan [°C]** |
| **DLGAP5** | **2** | chr14:54728600-54729400 | FW: 5’-ATTGGGGGGGAAAAATTATTAT-3’  RV: 5’-ACAACCTTAAACTACTATATTCAA-3’ | chr14:54728972-54729347 | **375** | **58** |
| **FCRL1** | **1** | chr1:156056300-156057300 | FW: 5’-GGAAGTAGAGTTTAAGTATGATTG-3’  RV: 5’-CTTAAAAAAATCCCTCAAATATT3’ | chr1:156056363-156056729 | **366** | **58** |
| **FCRL1** | **2** | chr1:156056300-156057300 | FW: 5’-GTTTTTTGGATTGTTTTTAATTGG-3’  RV: 5’-ACCCTTCTCAAATACCCTAAAAC-3’ | chr1:156056854-156057209 | **355** | **58** |
| **GPM6B** | **2** | chrX:13745500-13746000 | **-** | **-** | **-** | **-** |
| **MAGEA12** | **1** | chrX:151654100-151654600 | **-** | **-** | **-** | **-** |
| **MMP1** | **1** | chr11:102174350-102174700 | **-** | **-** | **-** | **-** |
| **SSX1** | **2** | chrX:47999200-47999950 | FW: 5’-GGTTTAAGTGATTTTTTAGTTGTG-3’  RV: 5’-TTTCTCTTTTCATTTTTCCTATCC-3’ | chrX:47999201-47999445 | **244** | **58** |
| **TPO** | **2** | chr2:1395918-1396518 | **-** | **-** | **-** | **-** |

**B) Nested primers for HRM.**

| **Gene** | **Probe #** | **Region [**UCSC**]** | **Nested primers** | | | |
| --- | --- | --- | --- | --- | --- | --- |
|  |  |  | **Sequence** | **Position [**UCSC**]** | **Amplicon size [bp]** | **Tan [°C]** |
| **DLGAP5** | **2** | chr14:54728600-54729400 | FW: 5’-GGATTTGATAATTGGGGTAAGTT-3’  RV: 5’-ACCATCCTCCCACCTCAA-3’ | chr14:54729097-54729323 | **226** | **58** |
| **FCRL1** | **1** | chr1:156056300-156057300 | FW: 5’-AGGTTAGGGATGGTATTTAGAGAT-3’  RV: 5’-TCACTAAAAAATTCTTCTTCCTTC-3’ | chr1:156056459-156056606 | **147** | **58** |
| **FCRL1** | **2** | chr1:156056300-156057300 | FW: 5’-TAGGGATTGATGGGGTTGTTA-3’  RV: 5’-CATTTTCAAATATCCCCCAAATT-3’ | chr1:156056960-156057157 | **197** | **53** |
| **GPM6B** | **2** | chrX:13745500-13746000 | FW: 5’-TTGGAGTGAGGAATTTTAATGAGT-3’  RV: 5’-AAAAAACACCCTTCCTTATTTAAA-3’ (Bio)  Seq: 5’-TTTATGTAGAAATAATTATA-3’ | chrX:13745128-13745286 | **158** | **53** |
| **MAGEA12** | **1** | chrX:151654100-151654600 | FW: 5’-TGGATGAAGTGATTGGTAGGA-3’  RV: 5’-TCTATCTTCTTCCAAAAATCAAT-3’ | chrX:151654325-151654480 | **155** | **56** |
| **MMP1** | **1** | chr11:102174350-102174700 | FW: 5’-TAGGGTAGAGGGTGGAATTATTAA-3’  RV: 5’-ACCAAACAACTTAACAAAAACAA-3’ | chr11:102174472-102174609 | **137** | **59** |
| **SSX1** | **2** | chrX:47999200-47999950 | FW: 5’-TGGTTTTTTTAAGTGTTGGGATTA-3’  RV: 5’-CTCAAATCCTTCCTATCAATTTCT-3’ | chrX:47999223-47999352 | **129** | **58** |
| **TPO** | **2** | chr2:1395918-1396518 | FW: 5’-TAGAGGTTGGATTGTATGTGGAT-3’  RV: 5’-CCAAAAACTTAATTACCCACCTT-3’ | chr2:1396040-1396242 | **202** | **58** |

C) QPCR primers.

| **Gene** | **Sequence** | **Amplicon size [bp]** | **Tan [°C]** |
| --- | --- | --- | --- |
|  |  |  |  |
| **GPM6B** | FW: 5’- GGTGCCCGTGTTTATGTTCTA -3’  RV: 5’- CACACAGATCTGCTCCACAC -3’ | **97** | **59** |
| **MAGEA12** | FW: 5’- CTTTAACCGCAGGGAACTCT -3’  RV: 5’- GTGTCTGTCGACTACATGGG -3’ | **100** | **59** |
| **FCRL1** | FW: 5’- CGACTCTGAGGTGCATTCTT -3’  RV: 5’- AGTGGAGCACAGATCAACAG -3’ | **104** | **59** |
